# Mixed Adenoneuroendocrine Carcinoma Is a Rare but Important Tumour Found in the Oesophagus

**DOI:** 10.1155/2016/9542687

**Published:** 2016-02-03

**Authors:** Mohammad Murad Kasim Kadhim, Marie Louise Jespersen, Hans Kristian Pilegaard, Marianne Nordsmark, Gerda Elisabeth Villadsen

**Affiliations:** ^1^Department of Surgery, Regional Hospital Horsens, Aarhus University Hospital, 8700 Horsens, Denmark; ^2^Department of Histopathology, Aarhus University Hospital, 8000 Aarhus C, Denmark; ^3^Department of Cardiothoracic and Vascular Surgery, Aarhus University Hospital, 8200 Aarhus N, Denmark; ^4^Department of Oncology, Aarhus University Hospital, 8000 Aarhus C, Denmark; ^5^Department of Hepatology and Gastroenterology, Aarhus University Hospital, 8000 Aarhus C, Denmark

## Abstract

Mixed adenoneuroendocrine carcinoma (MANEC) is a rare tumour of the gastrointestinal tract that consists of a dual adenocarcinomatous and neuroendocrine differentiation, each component representing at least 30% of the tumour. We report a case of a 68-year-old man who presented with two-month history of postprandial pain and vomiting. Gastric endoscopy revealed a polypoid mass in the lower part of the oesophagus. In contrast to the majority of these tumours, this biopsy was immunohistochemically positive for chromogranin A, and synaptophysin and Ki-67 index was 50% and the tumour was diagnosed as poorly differentiated neuroendocrine carcinoma of the oesophagus. The patient underwent surgery and lower oesophagus resection was performed. Based on the histopathology and immunohistochemistry of the tumour in the oesophagogastrectomy specimen, a mixed adenoneuroendocrine carcinoma (MANEC) was diagnosed. The objective of this case report is to advocate for the focus on the MANEC diagnosis as such patients need to be referred to a centre of excellence with expertise in NET tumours, to have the correct diagnostic work-up, treatment, and secondary diagnostic procedures performed at progression, as this will have paramount influence of the choice of treatment.

## 1. Introduction

Mixed adenoneuroendocrine carcinoma (MANEC) in the oesophagus is an extremely rare cancer diagnosis. However, correct diagnosis is important for guiding the choice of treatment and prognosis.

Planocellular and adenocarcinomas are the most common tumours of the oesophagus constituting 95% of primary epithelial carcinomas. Other rare tumours of the oesophagus include lymphomas, sarcomas, melanomas, and neuroendocrine tumours.

The purpose of this case report is to raise awareness of this diagnosis because it affects the treatment strategy and follow-up.

## 2. Case Report

A 68-year-old man was referred to our department for endoscopy with a 2-month history of postprandial pain and vomiting. The patient had a medical history of hypertension, type II diabetes, and hypercholesterolemia.

**Figure 1:**
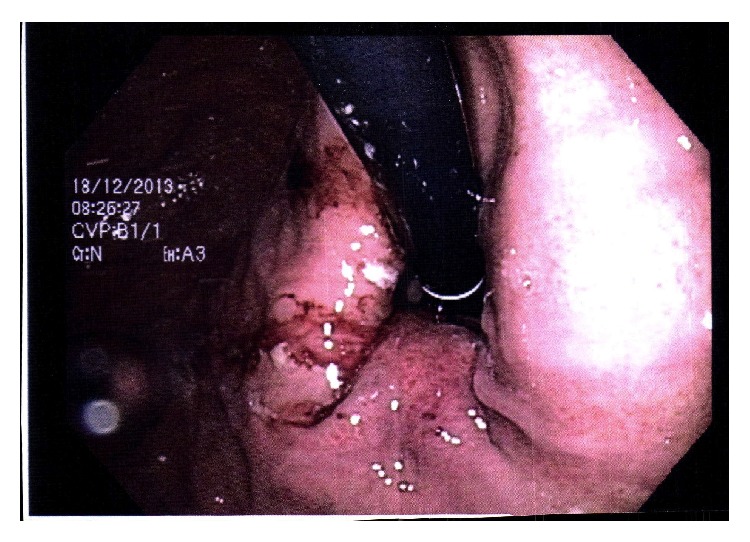
Polypoid mass in the distal oesophagus.

**Figure 2:**
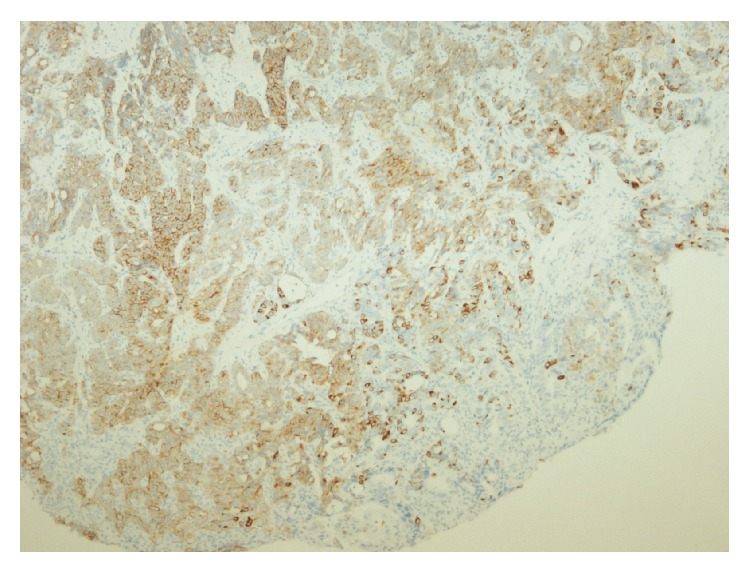
*Synaptophysin immunostaining* of tumour cells (brown colour).

Oesophagogastroduodenoscopy revealed a polypoid mass in the distal oesophagus, which was approximately 35 cm from the dental arch and extended to the gastrooesophageal junction (GEJ) (Figure 1). The histopathological examination of biopsies confirmed a diagnosis of neuroendocrine carcinoma (NEC). The tumour cells were positive for synaptophysin and focally positive for chromogranin A (Figure 2). The biopsy revealed varied proliferation rates by Ki-67 in hot spots up to 50%. There were 17 mitoses/10 high power fields (HPFs).

A PET-CT scan showed increased fluorodeoxyglucose (FDG) uptake, corresponding to the tumour in the distal oesophagus. Somatostatin receptor imaging using Gallium DOTANOC PET/CT revealed slightly positive uptake above liver, corresponding to a primary tumour in the oesophagus.

The patient underwent macroradical transthoracic oesophagus resection without oncological pretreatment. Histopathological examination of the specimen revealed a highly differentiated adenocarcinoma and neuroendocrine carcinoma (NEN G3) [1], alternating with invasion into the tunica muscularis and subserosa, which is compatible with mixed adenoneuroendocrine carcinoma (MANEC) of the composite/collision type with a total length of 95 mm. The mucosa was ulcerated, and there was detectable vascular invasion. The NEC component had metastasized to 7 of 30 lymph nodes.

## 3. Discussion

MANEC is a tumour that consists of two components, an adenocarcinoma and neuroendocrine tumour of the oesophagus, which is most often a neuroendocrine carcinoma. Referring to the WHO criteria, each component may represent at least 30% of the tumour [2]. MANEC is a rare type of tumour, and its occurrence in the oesophagus is extremely rare, making it a diagnostic challenge for clinicians. These patients should be referred to a highly specialized centre for neuroendocrine tumours, and pathologists with special expertise in neuroendocrine neoplasms should examine the histopathological specimen.

The use of somatostatin receptor scintigraphy (SRI) is appropriate for diagnosis and in follow-up of these tumours, but the use of SRI is not established. According to Ilett et al. [3] 37–71% of gastroenteropancreatic-neuroendocrine neoplasms are positive on somatostatin receptor scintigraphy.

The evidence for determining MANEC prognosis and treatment is limited [1, 4, 5]. MANEC in oesophagus is extremely rare, and there is no recommended therapeutic treatment strategy, and treatment is based on NEC studies.

Surgical resection is the primary treatment strategy in localized disease. Preoperative oncological treatment is experimental and may be used as a downstaging strategy for primarily nonresectable tumours. Adjuvant oncological treatment is poorly understood. According to the “Nordic guidelines for neuroendocrine neoplasms in 2014” [1] and to the European and North American guidelines for neuroendocrine neoplasms [6, 7], cisplatin or carboplatin and etoposide are recommended.

For patients with metastatic GEP NEC (gastroenteropancreatic-neuroendocrine neoplasms) and MANEC, rapid initiation treatment with palliative chemotherapy is important [8]. If the patient has a somatostatin receptor-positive tumour with strong uptake, experimental treatment with peptide radionuclide receptor therapy might be used as second- or third-line therapy.

In the case of recurrence or progression, it is important to assess patients with both FDG PET/CT and Gallium DOTANOC PET/CT. Furthermore, rebiopsy may be relevant because MANEC is a 2-component tumour with a potentially visible mixed response. The conclusion is that a diagnosis of MANEC should be considered when examining patients with a suspected tumour in the upper GI because a MANEC diagnosis has consequences for the treatment strategy and follow-up.
